# Secreted Human Amyloid Precursor Protein Binds Semaphorin 3a and Prevents Semaphorin-Induced Growth Cone Collapse

**DOI:** 10.1371/journal.pone.0022857

**Published:** 2011-07-29

**Authors:** Margaret H. Magdesian, Matthias Gralle, Luiz H. Guerreiro, Paulo José I. Beltrão, Milena M. V. F. Carvalho, Luís Eduardo da S. Santos, Fernando G. de Mello, Ricardo A. M. Reis, Sérgio T. Ferreira

**Affiliations:** 1 Institute of Medical Biochemistry, Program in Biochemistry and Cellular Biophysics, Federal University of Rio de Janeiro, Rio de Janeiro, Brazil; 2 Institute of Biophysics Carlos Chagas Filho, Program in Neurobiology, Federal University of Rio de Janeiro, Rio de Janeiro, Brazil; Columbia University, United States of America

## Abstract

The amyloid precursor protein (APP) is well known for giving rise to the amyloid-β peptide and for its role in Alzheimer's disease. Much less is known, however, on the physiological roles of APP in the development and plasticity of the central nervous system. We have used phage display of a peptide library to identify high-affinity ligands of purified recombinant human sAPPα_695_ (the soluble, secreted ectodomain from the main neuronal APP isoform). Two peptides thus selected exhibited significant homologies with the conserved extracellular domain of several members of the semaphorin (Sema) family of axon guidance proteins. We show that sAPPα_695_ binds both purified recombinant Sema3A and Sema3A secreted by transfected HEK293 cells. Interestingly, sAPPα_695_ inhibited the collapse of embryonic chicken (*Gallus gallus domesticus*) dorsal root ganglia growth cones promoted by Sema3A (K_d_≤8·10^−9^ M). Two Sema3A-derived peptides homologous to the peptides isolated by phage display blocked sAPPα binding and its inhibitory action on Sema3A function. These two peptides are comprised within a domain previously shown to be involved in binding of Sema3A to its cellular receptor, suggesting a competitive mechanism by which sAPPα modulates the biological action of semaphorins.

## Introduction

The amyloid precursor protein (APP) family members are ubiquitously expressed type I integral membrane proteins with relatively large extracellular domains and short intracellular domains. In the central nervous system (CNS), APP can be cleaved to generate the amyloid-β peptide (Aβ), which forms oligomers and amyloid plaques that accumulate in the limbic and cerebral cortices of Alzheimer's disease (AD) patients. It is now widely accepted that Aβ aggregates are the main neurotoxins that lead to synapse damage and neuronal dysfunction in AD [1], [2]. However, it is also possible that loss or disruption of physiological function(s) of APP and the underlying signal transduction pathways contribute to or initiate the pathological process in AD.

The physiological functions of APP have been subject of intense investigation and several lines of evidence suggest that APP regulates neurite outgrowth. Indeed, APP synthesis and axonal transport coincide with periods of axon elongation and synapse formation [3], [4]. The secreted extracellular domain of APP, sAPPα, acts as a growth factor for many cell types and promotes neuritogenesis in post-mitotic neurons [5]. Consistent with a physiological role for APP in neuron development [6], [7], secreted APP fragments and overexpression of transmembrane APP have both been shown to increase neurite outgrowth [8]–[10], whereas decreased APP expression alters process outgrowth [6], [11]–[12] and APP knock-out mice exhibit motor dysfunction and brain gliosis [6]. Moreover, recent data showed that a complex including APP, FE65 and an additional unknown protein is involved in neurite outgrowth at early stages of neuronal development [13]. However, the mechanisms by which APP regulates neurite outgrowth remain largely unknown.

Growing axons are guided to their targets by extracellular physical and molecular cues [14]. Such cues may have positive or negative effects on growth cone motility as they bind to surface receptors and trigger reorganization of cytoskeletal components underlying cone dynamics [15]. In developing tissues, growth cones simultaneously encounter multiple guidance cues and, therefore, the resulting cone behavior reflects integration of signaling by multiple cues [16]. Various molecules have been shown to have inhibitory activities on axonal growth during development [17] and to cause growth cone collapse or neurite repulsion *in vitro*. Among those molecules, semaphorins comprise the largest gene family of axonal growth inhibitory molecules [18].

Semaphorins consist of more than 20 secreted and membrane-bound proteins that are expressed in most tissues in a developmentally-regulated pattern. Semaphorins are dynamically expressed in the CNS during embryonic development and their expression is often associated with growing axons. Expression decreases with maturity and several observations indicate that in adult brain the expression of secreted semaphorins is sensitive to electrical activity and experience [18]. The functional role of semaphorins in guiding axon projections is well established and more recent evidence points to additional roles in a variety of cellular events, including neuronal migration [19]–[20], cell death by apoptosis [21], dendritic guidance and spine formation [22]–[23], axon pruning [24], axonal transport [25] and synaptic transmission [26]–[27]. Semaphorins exert the majority of their effects by binding to cognate receptor proteins through their extracellular domains. A common theme is that semaphorin-triggered signaling induces the rearrangement of the actin and microtubule cytoskeleton. Mutations in semaphorin genes are linked to several human neurological disorders, but their precise influence on the pathogenesis of such diseases remains to be determined.

With the goal of identifying novel neuronal ligands for APP, we have used phage display of a peptide library to identify peptides that bind to purified recombinant human sAPPα_695_ (the soluble ectodomain from the main neuronal isoform of APP, APP_695_). Using this approach, we isolated 2 heptapeptides that bind to sAPPα_695_ and are highly homologous to several members of the human semaphorin family. We show that sAPPα_695_ binds with high affinity to semaphorin 3A (Sema3A) and inhibits the collapse of chicken dorsal root ganglia (DRG) growth cones induced by Sema3A. These results provide the first evidence that APP regulates neurite outgrowth through interaction with members of the semaphorin family and open new avenues for characterization of novel physiological functions of APP.

## Materials and Methods

### Ethics statement

All experiments involving animals were approved by and carried out in accordance with the guidelines of the Institutional Animal Care and Use Committee of the Federal University of Rio de Janeiro (permit number IBCCF-035), and all efforts were made to minimize suffering.

### Reagents

Recombinant human sAPPα_695_ was produced and purified as described [28]. Anti-APP antibody was MAB348 from Chemicon (Temecula, CA) and recombinant Sema3A and anti-Sema3A were from R&D Systems (Minneapolis, MN). All peptides were synthesized by New England Peptides (Gardner, MA).

### Sema3A-AP conditioned medium

Human embryonic kidney 293 (HEK293) cells transfected with expression vectors for Sema3A conjugated to alkaline phosphatase (Sema3A-AP) [29] were kindly provided by Dr. Daniela R. Uziel (Federal University of Rio de Janeiro). Sema3A-AP-transfected or control non-transfected HEK293 cells were cultured in Dulbecco's Modified Eagle's Medium (DMEM; Invitrogen, Carlsbad, CA) supplemented with 10% fetal bovine serum (FBS, Invitrogen), 100 U/ml penicillin and 2 mM glutamine (Invitrogen) in a humidified 5% CO_2_ atmosphere at 37°C. Prior to experiments, cells were cultured in DMEM/10% FBS for 2 days and the conditioned control medium or medium containing Sema3A-AP were collected, filtered and stored at −20°C until used.

### Biopanning against sAPPα_695_

Screening of the phage display peptide library was performed as previously described [30]–[32]. Briefly, sAPPα_695_ was diluted to 0.1 µg/ml in PBS and 50 µl aliquots were incubated under constant shaking for 16 h in the wells of a 96-well plate at 4°C. The wells were then blocked with 0.5% bovine serum albumin (Sigma) in PBS for 1 h and 10 µl of a phage display library (PhD-C7C; New England Biolabs, Ipswich, MA) were added to each well. All further procedures were carried out exactly according to manufacturer's instructions. sAPPα_695_-binding phages were individually picked, amplified, and had their DNA sequenced. A heptapeptide with amino acid sequence LRSHPLG was expressed by 5 out of 28 sAPPα_695_-binding clones. Another peptide with amino acid sequence TFASVMT was expressed by an additional sAPPα_695_-binding clone. The amino acid sequences of the two peptides were compared with protein sequences deposited in several data banks (NCBI) using BLAST [33].

### Binding of Sema3A to sAPPα_695_

Fifty or 100 ng of sAPPα_695_ (as indicated in “Results”) in 50 µl of PBS were added to wells in a 96-well plate and were incubated overnight at 4°C. Wells were then washed with PBS and blocked for 2 h with 1% BSA in PBS. Different concentrations of purified recombinant Sema3A in PBS containing 0.5% BSA or Sema3A-AP-containing conditioned medium were added to the wells and incubated overnight at 4°C with agitation. The wells were washed six times with PBS, incubated with anti-Sema3A for 1 h, washed, incubated with horseradish peroxidase-conjugated secondary antibody for 1 h and developed with SuperSignal ELISA Femto Substrate (Pierce, Rockford, IL). When Sema3A-AP was used, binding was directly evaluated by determining alkaline phosphatase activity with *p*-nitrophenyl phosphate (Sigma) as substrate.

In another set of experiments, sAPPα_695_ was biotinylated using the EZ-LinkSulfo-NHS-Biotin reagent (Pierce, Rockford, IL) and added at different concentrations to wells that had been previously coated with 50 ng recombinant Sema3A and blocked with 1% hen egg-white lysozyme (Sigma) instead of BSA, in order to avoid the binding of sAPPα_695_ to BSA. Nonspecific binding was determined in the presence of 100-fold excess non-biotinylated sAPPα_695_. The wells were washed six times with PBS, incubated with horseradish peroxidase-conjugated streptavidin (Sigma) for 1 h and developed with SuperSignal ELISA Femto Substrate. Each experiment was performed in triplicate.

### Pull-down and immunoprecipitation experiments

Streptavidin-coupled Dynabeads (Invitrogen) were incubated with biotinylated sAPPα_695_ (bAPP) for 1 h, washed with PBS and then incubated for 2 h with Sema3A-AP-containing conditioned medium or control medium (conditioned by non-transfected HEK293 cells). After washing the beads, binding was evaluated by measuring phosphatase activity using *p*-nitrophenyl phosphate. Alternatively, protein A-Sepharose beads (Amersham GE) were incubated at 4°C with anti-Sema3A plus 120 µl of Sema3A-AP-containing conditioned medium or 120 µl of control medium and 300 ng of bAPP. Immunoprecipitation was conducted in the presence or in the absence of the ARSHPAM and LTASLLI peptides (as described in “Results”). The beads were then washed six times with PBS containing 0.1% Nonidet P40 (Sigma) and the immunoprecipitated material was separated by SDS-PAGE. Protein bands were transferred to nitrocellulose and the membrane was probed with horseradish peroxidase-conjugated streptavidin and developed with SuperSignal Femto Substrate.

### Growth cone collapse assay

Dorsal root ganglia (DRG) were dissected from E7 chick embryos as previously described [34]. Ganglia explants were cleaned, cut in halves or quarters and plated at low density on poly-L-lysine- and laminin-coated 4-well tissue culture dishes (Nalge Nunc, Rochester, NY). DMEM containing 10% fetal calf serum with gentamycin (10 µg/ml) was supplemented with 20 ng/ml NGF 2.5S (Sigma). For immunohistochemistry experiments, the medium was additionally supplemented with 75 nM sAPP, 0.8 nM Sema3A and/or 75 nM of the ARSHPAM and LTASLLI peptides (see “Results”) before plating. Cultures were kept for 24 h at 37°C in a humidified 5% CO_2_ incubator. Samples were fixed with 4% paraformaldehyde, permeabilized with 0.25% Triton X-100 for 5 min and labeled for neurofilaments [35] using mouse monoclonal antibody 3A10 (Hybridoma Bank, University of Iowa) and Alexa488-conjugated rabbit anti-mouse IgG (Invitrogen). For quantification of growth cone morphology by phase contrast, Sema3A, sAPP and/or peptides were added 30 min before fixation.

Randomly selected fields at the border of the ganglia were imaged either by fluorescence or by phase contrast microscopy on a Zeiss Axiovert microscope. All images were collected using 20× or 40× objectives, and the contrast in phase contrast images was automatically adjusted using NIH ImageJ software [36]. For each image, the number of axonal endings was scored by one of two different experimentators as either a growing growth cone with lamellipodia and filopodia or a collapsed cone (a tapered axonal terminal without spread lamellipodia or less than three filopodia). Seven independent experiments were performed with different ganglion cultures. The percentage of collapsed growth cones for each ganglion and means ± SEM for each experimental condition were calculated (control: n = 7 ganglia; sAPPα: n = 4; peptides: n = 9; Sema3A: n = 19; Sema3A+sAPPα: n = 15; Sema3A+sAPPα+peptides: n = 9).

The minimum adequate generalized linear binomial model [37], [38] showed significant differences (*p*<0.001) between results obtained in one group of conditions (control, sAPPα and Sema3A+sAPPα) and another group of conditions (Sema3A and Sema3A+sAPPα+peptides). Furthermore, Bonferroni-corrected *p* values for multiple comparisons between conditions are indicated in the legend to the figure.

## Results

### Semaphorins as sAPPα ligands

**Figure 1:**
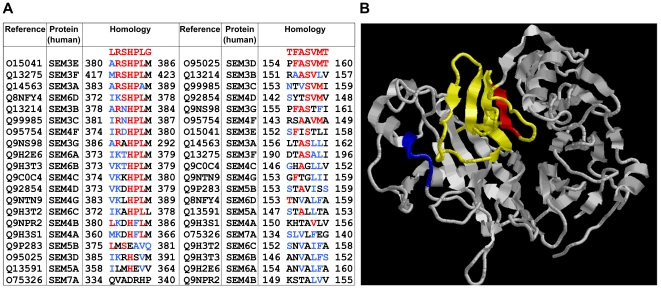
sAPPα-binding peptides selected by phage display are homologous to members of the human semaphorin family. (A) Sequence alignment of the LRSHPLG and TFASVMT peptides with members of the human semaphorin family; identical residues are shown in red and conservative amino acid replacements are in blue. Sequence alignment was performed using ClustALL [56] (B) Ribbon representation of the structure of the receptor binding module of Semaphorin 3A. The amino acid sequences homologous to the LRSHPLG (blue; ARSHPAM) and TFASVMT (red; LTASLLI) peptides are highlighted. Residues involved in receptor specificity [39] are shown in yellow and red. The figure was generated using RasMol and the atomic coordinates for Sema3A (Protein Data Bank accession code 1Q47; ref. 40).

In order to identify putative sAPPα ligands, we initially screened a phage display peptide library and selected clones that bound specifically to sAPPα_695_. Five out of 28 individually picked sAPPα-binding clones contained the peptide sequence LRSHPLG. An additional sAPPα-binding clone comprising amino acid sequence TFASVMT was isolated. The sequences of both peptides were then compared to human protein sequences in the NCBI databank using BLAST [33]. This analysis revealed that both peptides presented significant sequence homologies to several members of the human semaphorin protein family (Fig. 1A). Interestingly, the TFASVMT peptide is homologous to a region of semaphorin 3A (Fig. 1B) that has been shown to be important for receptor recognition [39], [40], while the region that is homologous to the LRSHPLG peptide lies very close to that same important site. These initial results suggested that sAPPα binds to semaphorins at a domain that is important for receptor recognition and biological activity.

### sAPPα binds to Sema3A

**Figure 2:**
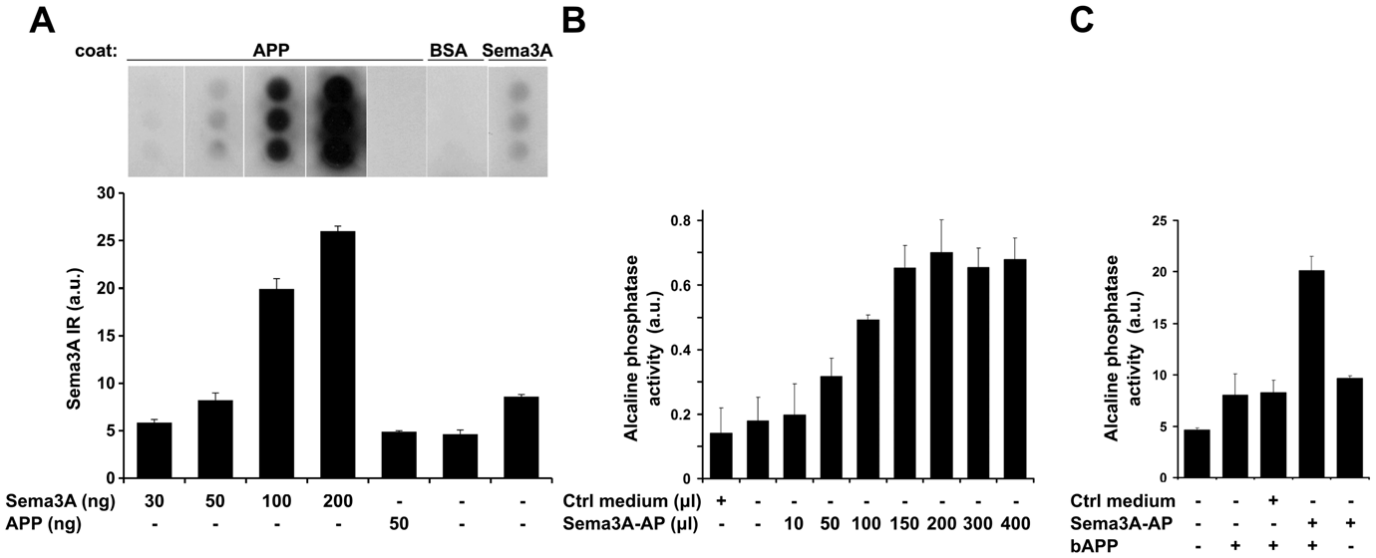
Semaphorin 3A specifically binds sAPPα. (A) Wells in a 96-well plate were coated with either 50 ng sAPPα_695_ (APP), 1 µg BSA or 50 ng Semaphorin 3A (Sema3A), blocked with 1% BSA and overlayed with different concentrations of Sema3A or sAPPα_695_ (as indicated below the lanes). After washing, binding was determined by probing with anti-Sema3A antibody. The graph shows concentration-dependent binding of Sema3A to sAPPα_695_ by densitometric quantification of the immunodots using NIH Image J. Bars represent averages of three independent experiments performed in triplicate each. The blot illustrates a representative experiment. (B) Wells in a 96-well plate were coated with 100 ng sAPPα_695_ (APP), blocked with 1% BSA and incubated with increasing volumes of conditioned medium containing alkaline phosphatase-conjugated Semaphorin 3A (Sema3A-AP). After washing, binding was determined by measuring alkaline phosphatase activity. Bars represent averages of three independent experiments performed in triplicate. (C) Streptavidin-coated beads were incubated with biotinylated sAPPα_695_ (bAPP) and Sema3A-AP-conditioned medium or control (non-transfected) medium. After washing, binding was evaluated by measuring alkaline phosphatase activity associated with the beads. Bars represent averages of three independent experiments.

In order to investigate the interaction between sAPPα and semaphorin, we performed direct *in vitro* binding assays using semaphorin3A of different origins. First, we immobilized sAPPα_695_ on microplate wells and added different concentrations of purified Sema3A (R&D Systems; the only semaphorin family recombinant protein commercially available at the time) or BSA (as a negative control) and probed with anti-Sema3A antibody. This assay revealed that Sema3A binds specifically to sAPPα (Fig. 2A). Next, we performed a similar experiment using conditioned medium from HEK293 cells transfected with Sema3A conjugated to alkaline phosphatase (Sema3A-AP). Addition of increasing concentrations of conditioned medium containing Sema3A-AP caused a dose-dependent binding of semaphorin to sAPPα (Fig. 2B), while no specific binding was detected when control medium (not containing Sema3A) was used. Furthermore, Sema3A-AP incubated with biotinylated sAPPα (bAPP) could be specifically precipitated by streptavidin-coated beads (Fig. 2C). Taken together, these results demonstrate binding of sAPPα to Sema3A using three different approaches.

### sAPPα modulates semaphorin function

**Figure 3:**
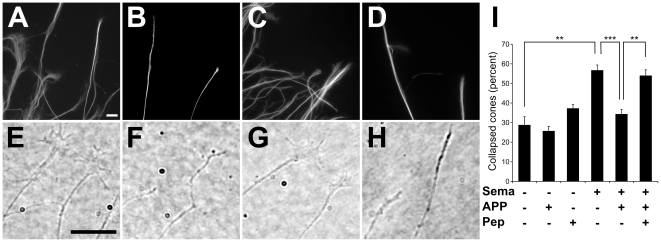
sAPPα modulates the biological activity of Sema3A. Growth cone morphology was examined in control chick DRG explants (A,E) and in explants challenged for 30 minutes with 0.8 nM Sema3A (B,F), Sema3A+75 nM sAPPα_695_ (C,G) or Sema3A+sAPPα_695_+75 nM each of peptides ARSHPAM and LTASLLI (D,H). After fixation and neurofilament immunostaining, growth cone morphology was examined using fluorescence (upper panels) or phase contrast microscopy (lower panels). Scale bars: 10 µm. Representative images (A–H) illustrate on average two growth cones, but more than 700 growth cones were evaluated in each experimental condition. Panel I shows results of quantitative analysis of the percentage of collapsed growth cones (as determined from phase contrast images) in each experimental condition. Bars correspond to means ± SEM of different ganglia. Each experimental condition was replicated 3–6 times in independent experiments using different DRG cultures. Asterisks represent statistically significant differences (**p<0.01; ***p<0.001; ANOVA followed by Bonferroni post-hoc test).

Because sAPPα was found to interact with Sema3A, we asked whether nanomolar concentrations of sAPPα might interfere with Sema3A-induced collapse of DRG axonal growth cones. As expected, DRG neurons showed extensive neurites after 24 hours in culture in the presence of NGF [41] (Fig. 3A,E). Addition of 0.8 nM Sema3A caused massive growth cone collapse (Fig. 3B,F,I). Remarkably, co-application of 75 nM sAPPα_695_ together with Sema3A inhibited the collapse of most growth cones (Fig. 3C,G,I). To further confirm the specificity of binding and the identity of the putative binding site on Sema3A, we synthesized the peptides within Sema3A that are homologous to the peptides identified by phage display. As shown in Fig. 1A, amino acid sequences ARSHPAM and LTASLLI in Sema3A are homologous to the LRSHPLG and TFASVLT peptides, respectively. Interestingly, both ARSHPAM and LTASLLI peptides blocked the inhibition by sAPPα of Sema3A-induced growth cone collapse (Fig. 3D,H,I), suggesting that inhibition is indeed due to direct and specific interaction between sAPPα and Sema3A at the binding site defined by the two peptides.

While our data do not permit calculating a simple affinity constant between sAPPα and Sema3A due to the possibility of higher-order interactions between the proteins or additional binding partners in living cells, it is possible to estimate an upper limit for the dissociation constant based on the finding that the biological activity of 0.8 nM Sema3A is completely blocked by 75 nM sAPPα (Fig. 3I). Assuming that Sema3A and sAPPα bind in a 1∶1 stoichiometry and that ≤10% of Sema3A remains unbound in the presence of this concentration of sAPPα, the dissociation constant K_d_ is ≤8·10^−9^ M. Presence of higher order binding complexes would imply an even higher affinity between the two proteins.

## Discussion

Several studies suggest roles for APP in neurite outgrowth and synaptogenesis, but the detailed molecular interactions that mediate the functions of APP during neuronal development are largely unknown (for a review, see ref. 5). A major current unknown is the identity of the neuronal receptors that mediate the physiological functions of sAPPα. Identification of such receptors would provide insight into the physiological roles of sAPPα and, possibly, into mechanisms of pathogenesis in AD. Using phage display of peptide libraries [30]–[32], we have now identified a number of peptides that bind sAPPα and are homologous to neuronal receptors putatively involved in sAPPα interactions. We report here on two heptapeptides that are homologous to regions in the extracellular domains of several members of the human semaphorin (Sema) family of proteins. Based on this homology, we set out to investigate the interaction between sAPPα_695_ and Sema3A.

Sema3A is a secreted protein that signals growth cone collapse, chemorepulsion and neuronal apoptosis during early CNS development [18]. We found that recombinant human sAPPα_695_ binds to Sema3A and inhibits the collapse of chicken DRG growth cones triggered by Sema3A. Interestingly, synthetic peptides corresponding to the amino acid sequences in Sema3A homologous to the peptides identified by phage display (ARSHPAM and LTASLLI) blocked both the binding of sAPPα_695_ to Sema3A and the inhibitory effect of sAPPα on Sema3A-induced growth cone collapse. These results suggest that sAPPα binds to a site near the region defined by the ARSHPAM and LTASLLI amino acid sequences in Sema3A.

In harmony with our findings, it has been shown that the extracellular domain of sAPPα_695_, containing the amino acid sequence RERMS (amino acid residues 328–332), induces neurite outgrowth and neuronal survival [42]. Using an affinity column containing bound RERMS peptide, collapsin response mediator protein 2 (CRMP-2) was identified as a potential sAPPα_695_ receptor [43]. CRMP-2 is an important downstream intracellular mediator of Sema3A signaling and the high affinity binding of CRMP-2 to sAPPα_695_ suggests a functional interaction of sAPPα with the semaphorin receptor complex. Although it is tempting to speculate that interaction with Sema3A may involve the RERMS domain of sAPPα, this question remains open for further investigation.

A recent study showed that sAPPα and full-length membrane-bound APP can each interact biochemically and functionally with integrin beta I to mediate neurite outgrowth [44], while sAPPα also directly binds to and dissociates dimers of full-length membrane-bound APP [45]. The signaling pathways of semaphorins and integrins are closely linked, and integrin activation/inhibition is central to signaling of different members of the semaphorin family [46]. Of particular interest, Sema3A inhibits integrin beta 1 signaling and promotes growth cone collapse [47]. Significantly, inhibition of integrin beta I function was also shown to block the outgrowth-enhancing effects of sAPPα [44]. Our data indicate that sAPPα may also interact with other members of the semaphorin family in addition to Sema3A, suggesting that sAPPα/semaphorin complexes may regulate multiple aspects of neuronal development and function.

It was recently shown that APP interacts with contactins 3 and 4 and associates with neuronglia cell adhesion molecule (NgCAM) in retinal axons growing into the optic tectum of embryonic chick brain [48]. Functional assays revealed regulatory effects of both APP and contactin 4 on NgCAM-dependent growth of cultured retinal axons. However, the mechanisms of binding of APP to those ligands are still unclear, and it is unknown whether other ligands or receptors are involved in the interactions.

While a role for Sema3A in axon guidance during development has emerged from several studies, its functions in the adult nervous system are not as well understood. Studies concerning the localization of secreted semaphorins revealed that Sema3A is transported in secretory vesicles that move towards axonal terminals, and this rapid microtubule-dependent transport is responsive to neuronal activity [49]. In contrast, in dendritic compartments, vesicles containing Sema3A appear more or less stationary. These data support the idea that activity-dependent release of secreted semaphorins occurs at synaptic sites *in vivo*. Interestingly, APP is also transported in vesicles along axons by a fast, kinesin-dependent anterograde transport mechanism [50]. Moreover, overexpression of APP in *Drosophila* larvae induces impairment of axonal transport of vesicles together with abnormalities in synaptic plasticity [51]. APP has recently been shown to interact with diverse synaptic proteins [52]–[55], and semaphorin-APP interactions may also play a role in the regulation of synaptic transmission and plasticity.

In conclusion, results presented here show that secreted APP interacts with different members of the semaphorin family, revealing a role for APP in regulating axon growth and guidance. These findings may pave the way for elucidation of novel functions of APP in normal CNS development as well as in physiological and pathological mechanisms of plasticity.
